# Differential expression pattern of *Vago* in bumblebee (*Bombus terrestris*), induced by virulent and avirulent virus infections

**DOI:** 10.1038/srep34200

**Published:** 2016-09-29

**Authors:** Jinzhi Niu, Ivan Meeus, Guy Smagghe

**Affiliations:** 1 Key Laboratory of Entomology and Pest Control Engineering, College of Plant Protection, Southwest University, Chongqing 400716, China; 2 Department of Crop Protection, Faculty of Bioscience Engineering, Ghent University, Coupure Links 653, B-9000 Ghent, Belgium

## Abstract

Viruses are one of the main drivers of the decline of domesticated and wild bees but the mechanisms of antiviral immunity in pollinators are poorly understood. Recent work has suggested that next to the small interfering RNA (siRNA) pathway other immune-related pathways play a role in the defense of the bee hosts against viral infection. In addition, Vago plays a role in the cross-talk between the innate immune pathways in *Culex* mosquito cells. Here we describe the Vago orthologue in bumblebees of *Bombus terrestris*, and investigated its role upon the infection of two different bee viruses, the virulent Israeli acute paralysis virus (IAPV) and the avirulent slow bee paralysis virus (SBPV). Our results showed that *Bt*Vago was downregulated upon the infection of IAPV that killed all bumblebees, but not with SBPV where the workers survived the virus infection. Thus, for the first time, *Vago/Vago-like* expression appears to be associated with the virulence of virus and may act as a modulator of antiviral immunity.

Insects and other invertebrates lack an adaptive immune system, which indicates that the innate immunity in these species is critical to modulate viral infections. Based on the mechanism to tackle virus in insects, the antiviral innate immune pathways can be generally grouped into two strategies of either nucleotide-based [as RNA interference (RNAi)] or protein-based [as Toll, Immune Deficiency (Imd) and the Janus kinase/signal transducer and activator of transcription pathway (JAK/STAT)]^1^,^2^. The small interfering RNA (siRNA) pathway (i.e., sub-pathway of RNAi) is the most prominent example for the nucleotide-based antiviral strategy in insects. During viral infection, specifically in RNA viruses, virus-related dsRNAs are generated from viral dsRNA replication intermediates, the viral genome itself, and/or viral transcriptions^3^. These virus-related dsRNAs are processed into 21~22 nt siRNAs by Dicer, and then these siRNAs are loaded onto Argonaute (Ago) forming the RNA-induced silencing complex (RISC) with other proteins^4^. One of the strands of siRNAs is selected and serves as a sequence-specific guide to cleave target viral mRNA (or genome) by complementary binding^5^. In mammals, these virus-derived long dsRNAs generally induce a protein-based innate antiviral immune response called the interferon response to control viral infection, which supplants the insect antiviral response of the siRNA pathway^6^. As a protein-based innate antiviral immune pathway, JAK/STAT has also been reported to combat various viruses in insects^7^,^8^,^9^. This pathway was discovered from the role of interferon in the control of immune response in vertebrates, and is now recognized to play a very important role in the regulation of both innate immune and adaptive immune systems^10^,^11^. In insects, the JAK/STAT pathway is generally initiated by the ligand unpaired (Upd) binding to the transmembrane receptor Domeless (Dome) which is a distant homolog of the vertebrate type I cytokine. The conformational change of Dome after Upd binding leads to the self-phosphorylation of the Janus kinase Hopscotch (Hop); however, the homologue of Upd was not identified in bees^12^,^13^. The activated Hop phosphorylates Dome, thereby forming docking sites for the cytoplasmic STATs. The recruitment of STAT by these docking sites enables Hop to phosphorylate STAT which leads to its dimerization. Subsequently, the STAT dimers translocate to the nucleus where they activate transcription of specific effectors to control viruses^14^. Intriguingly, the siRNA pathway communicates with the JAK/STAT pathway during viral infection in mosquito cells through Vago^7^,^15^,^16^. Briefly, upon viral infection, the upregulation of *Dicer-2* of the siRNA pathway leads to the activation of *Vago* transcription, which increases the level of secreted Vago. Subsequently, Vago induces the JAK/STAT antiviral immunity in a manner which is similar to mammalian interferon^7^,^15^. However, the role of Vago in other insects during viral infections is rarely known^7^,^15^,^17^.

Viruses, being often transmitted between domesticated and wild bees^18^,^19^,^20^,^21^, are proven to be one of the drivers of bee colony declines^22^,^23^. Bee viruses are mainly from the families of *Dicistroviridae* and *Iflaviridae* in the order of *Picornavirales*, which are non-enveloped small icosahedral virions, covering a positive sense single stranded RNA genome. In wild bumblebees, Israeli acute paralysis virus (IAPV) from *Dicistroviridae* and slow bee paralysis virus (SBPV) from *Iflaviridae* have been detected^18^,^19^,^20^,^21^, but symptoms of infection have not yet been reported. In our previous study^24^, IAPV replicates very fast and acts as a very virulent virus in bumblebees while SBPV also replicates fast but induces no mortality after injection. Furthermore, both IAPV and SBPV infections could induce the expression of *Dicer-2* in *B. terrestris*. IAPV but not SBPV infection triggered the production of predominantly 22 nt-long virus-derived siRNAs. However, the silencing of *Dicer-2* by RNAi did not result in an altered genome copy number of IAPV and SBPV^24^. In both honeybees and bumblebees, non-specific dsRNA can also induce a noticeable antiviral activity^25^,^26^. Indeed in honeybee, genes from immune pathways like RNAi, Toll and JAK/STAT, showed different expressions upon bee viral infections^27^. Thus, it is speculated that other immune pathways could be important in the control of viral infections. Here we suggest that the antiviral response in bumblebees goes beyond the siRNA pathway and the communication between the siRNA pathway and others could play an important role. Therefore, in this study, we described the Vago orthologue in bumblebees of *Bombus terrestris*, and then investigated its role as a possible communication gene between the siRNA pathway and JAK/STAT pathway. *B. terrestris* is one of the most numerous bumblebee species in Europe and important for the pollination of wild flowers and also many crops in agriculture. The study of immunity in bees is different from studies in model insects like *Drosophila* and mosquito. Indeed different bees, ranging from solitary to social bees, all have a rather small immune genes repertoire^12^,^28^,^29^. This lower immune repertoire in bees, with a different degree of sociality, indicates the evolution of bee immunity predates the evolution of sociality^12^,^28^. Here, we used *B. terrestris* as a model for a primary social insect to see how they respond to different viral infections, specifically IAPV as virulent infection and SBPV as avirulent infection.

## Results

### The orthologue of Vago in *B. terrestris*

**Figure 1:**
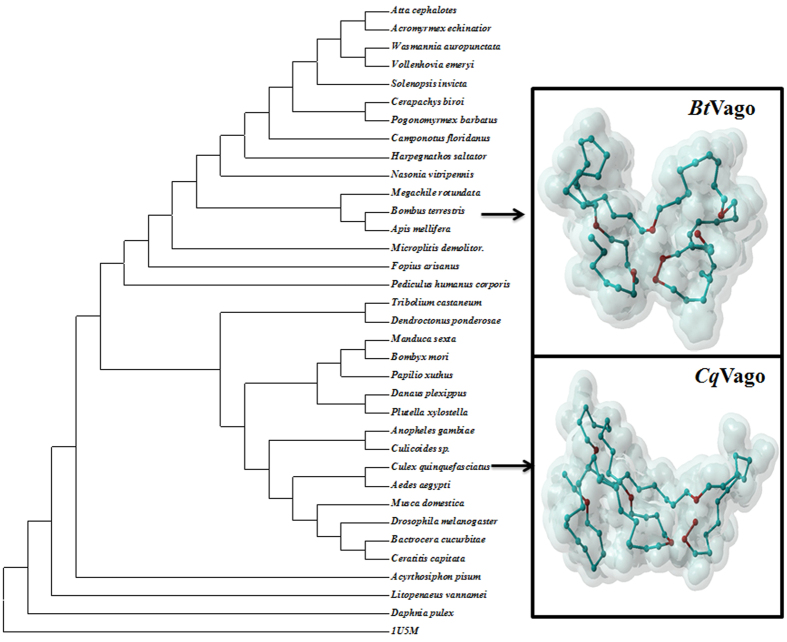
Phylogenetic analysis of Vago-like protein sequences and proposed 3D structures of *Bt*Vago and *Cq*Vago based on 1U5M. MEGA 6.0^37^, was used to construct the phylogenetic tree of Vago-like proteins through Maximun Likelihood. The conserved amino acids from chosen sequences of each species were used (Supplementary file: S1). The number of Bootstrap replications was 500 to test the phylogeny. The model of LG + G was adapted according to the model test of all input sequences. The proposed protein 3D structures of *Culex quinquefasciatus* Vago and *Bombus terrestris* Vago-like proteins, was constructed based by SWIS-MODEL through the template from Protein Data Bank (PDB code 1U5M). The amino acids in red shows conserved cysteine residues of each Vago were alignment with the 1U5M during the model constructions.

From the genome of *B. terrestris*, a protein sequence of 153-aa presenting the similar character with *Cx*Vago (Vago of *Culex quinquefasciatus*: XP_ 001842264.1) was obtained. This protein sequence showed eight conserved cysteine residues which form a von Willebrand factor C-domain (VWC) (Supplementary file: Figure S1). Phylogenetic analysis of Vago-related protein sequences revealed that insect Vago mainly followed the taxonomy (Fig. 1), indicating that this protein sequence obtained from *B. terrestris* was closely related with honeybee Vago. Based on the VWC domain as a template from Protein Data Bank (PDB code 1U5M), the 3D protein structure of *Bt*Vago and *Cx*Vago were constructed by Swiss-model (http://swissmodel.expasy.org/interactive accessed on: April 22, 2015), while only seven conserved cysteine residues of each Vago were alignment with the 1U5M (Fig. 1). Therefore, this orthologue of Vago/Vago-like from *B. terrestris* was named as proposed *Bt*Vago (XP_003399812.1). SignalP 4.1^30^ analysis of the *Bt*Vago amino acid sequence indicated a signal peptide cleavage site between amino acids 16 and 17 (Supplementary file: Figure S2). This suggests that *Bt*Vago may also be secreted as described in mosquito to present an interferon-like antiviral function^7^,^15^. Since it is difficult to prove this functions *in vivo* and because of the lack of cell line of bumblebees at this moment, the biological evidence to prove that *Bt*Vago may also be secreted and to test the overexpression of *Bt*Vago in effecting viral titers, awaits further studies. In order to identify the possible promoter region responsible for *Bt*Vago activation, an ~2 kb upstream from the transcription start site in the 5′ regions was analyzed by PROMO. The results indicated the presence of a NF-κB biding site in the *Bt*Vago promoter region (Supplementary file: Table S1).

### *Bt*Vago was downregulated upon infection with IAPV but not SBPV

**Figure 2:**
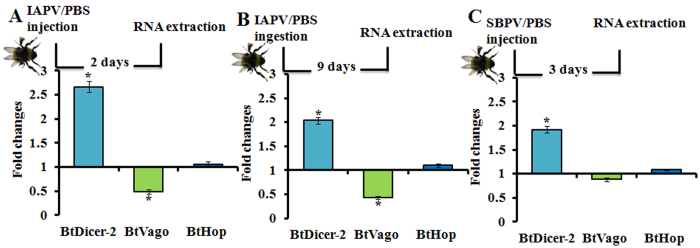
Fold changes of *Bt*Dicer-2, *Bt*Vago, and *Bt*Hop upon viral infections in comparison with controls (PBS). (**A**) IAPV infection was inoculated by injection. Two days after IAPV injection, the RNA samples were collected for gene expression analysis, PBS injected samples were collected as control. (**B**) IAPV infection was inoculated by ingestion (feeding). Five days after IAPV ingestion, the RNA samples were collected for gene expression analysis, PBS ingested samples were collected as control. (**C**) SBPV infection was inoculated by injection. Three days after SBPV injection, the RNA samples were collected for gene expression analysis, PBS injected samples were collected as control. Each treatment included eight biological replicates. The fold changes were equal to division of relative expression of each gene in virus infected samples by relative expression of the very gene in control samples. The relative expression of each gene was calculated based on internal reference gene *PPIA*. Each bar errors was represented by standard error of mean. The asterisk (*) represent statistical significant difference of mean (*p* < *0.05*).

*In silico* analysis above provided some evidences of the presence of a functional *Bt*Vago, thus we wondered whether the viral infections, such as virulent (IAPV) and avirulent (SBPV) infections, could influence the expression of *Bt*Vago. Upon viral infection in mosquito, the siRNA pathway can induce the expression of Vago through a Dicer-2 dependent manner, which further lead to the activation of JAK-STAT pathway^7^,^15^. Therefore, we chose the time points with *Bt*Dicer-2 upregulation induced by the infection of IAPV or SBPV based on our previous study^24^, to study the expression of *Bt*Vago. In contrast to mosquito and honeybee showing an upregulation of *Vago* upon viral infections^7^,^15^,^31^, our results (Fig. 2A,B) showed that the expression of *Bt*Vago was downregulated upon the infection of IAPV (both inoculation ways: injection (t-test: t = −3.773, df = 8.5, *p* = *0.005*) and ingestion (t-test: t = 2.211, df = 14, *p* = *0.044*) when the expression of *Bt*Dicer-2 was significantly upregulated with the IAPV infection. Conversely, the expression of *Bt*Vago was not changed upon the infection of SBPV when the expression of *Bt*Dicer-2 was significantly upregulated (Fig. 2C).

### Silencing of *Bt*Vago downregulated *Bt*Dicer-2 expression upon IAPV infection but downregulated *Bt*Hop expression upon SBPV infection

**Figure 3:**
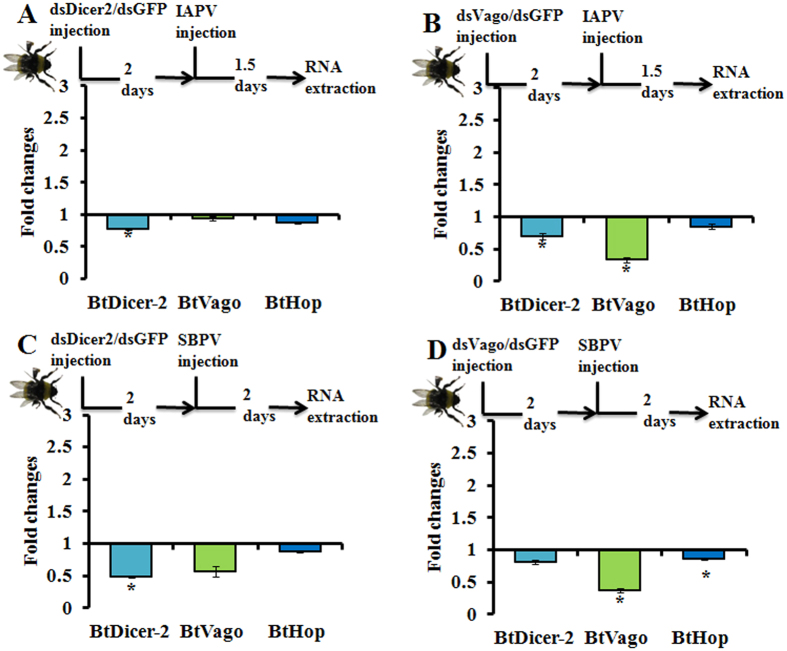
Fold changes of *Bt*Dicer-2, *Bt*Vago, and *Bt*Hop upon viral infections with pre-silencing of *Bt*Dicer-2 or *Bt*Vago in comparison with controls. (**A**) IAPV infection with *Bt*Dicer-2 pre-silencing. DsDicer2 was injected to silence *Bt*Dicer-2, two days later, IAPV was injected to inoculate bees. Subsequently, RNA was collected post 1.5 days injection of IAPV for measuring gene expressions (n = 18). (**B**) IAPV infection with *Bt*Vago pre-silencing. DsVago was injected to silence *Bt*Vago, two days later, IAPV was injected to inoculate bees. Subsequently, RNA was collected post 1.5 days injection of IAPV for measuring gene expressions (n = 9). (**C**) SBPV infection with *Bt*Dicer-2 pre-silencing. DsDicer2 was injected to silence *Bt*Dicer-2, two days later, SBPV was injected to inoculate bees. Subsequently, RNA was collected post two days injection of SBPV for measuring gene expressions (n = 8). (**D**) SBPV infection with *Bt*Vago pre-silencing. DsVago was injected to silence *Bt*Vago, two days later, SBPV was injected to inoculate bees. Subsequently, RNA was collected post two days injection of SBPV for measuring gene expressions (n = 15). We used dsGFP and PBS injections as controls for dsRNA and viral injections, respectively, for each treatment. The fold changes were equal to division of relative expression of each gene in samples of viral infections and pre-gene silencing by relative expression of the very gene in control samples. The relative expression of each gene was calculated based on internal reference gene *PPIA*. Each bar errors was represented by standard error of mean. The asterisk (*) represent statistical significant difference of mean (*p* < *0.05*).

Next, we wondered whether the depletion of *Bt*Dicer-2 or *Bt*Vago could interfere with each other’s expression upon the stress of viral infections. The results showed that the silencing of *Bt*Dicer-2 upon both viral infections did not influence the expression of *Bt*Vago and *Bt*Hop (Fig. 3A,C). Indeed, in a RNAi-of-RNAi experiment, the pre-silencing of *Bt*Dicer-2 impacted the later-silencing of peptidylprolyl isomerase A (PPIA) suggesting that this minor drop had functional implication (Niu *et al*., 2016), thus the non-detection of an effect on *Bt*Vago and *Bt*Hop to virus is probably not a consequence of the somewhat low silencing efficiency of RNAi^24^. The silencing of *Bt*Vago upon IAPV but not SBPV infection significantly decreased the expression of *Bt*Dicer-2 (t-test: t = 4.009, df = 17.3, *p* = *0.001*) (Fig. 3B), while the silencing of *Bt*Vago upon SBPV but not IAPV infection showed a significant downregulation of *Bt*Hop (t-test: t = 2.153, df = 18, *p* = *0.045*) (Fig. 3D). In the bumblebees without SBPV or IAPV infection, the silencing of *Bt*Dicer-2 or *Bt*Vago showed no effect to each other’s expression. Additionally, the silencing of *Bt*Vago presented a downregulation of *Bt*Hop, again suggesting an association between *Bt*Vago and JAK/STAT pathway (Supplementary file: Figure S3). In summary, these results indicated that the disruption of *Bt*Vago translation may interfere with the expression of *Bt*Dicer-2 upon IAPV infection or the expression of *Bt*Hop upon SBPV infection, respectively.

### Silencing of *Bt*Vago did not influence IAPV and SBPV genome copy numbers

**Figure 4:**
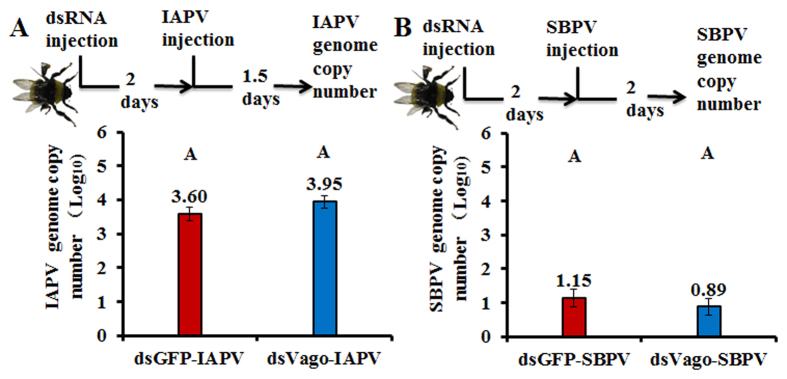
Genome copy numbers of viruses upon silencing of *Bt*Vago. (**A**) IAPV: DsVago (n = 9) was injected to silence *Bt*Vago, two days after injection, IAPV was injected to inoculate bees. Subsequently, RNA was collected post 1.5 days injection of IAPV for measuring viral genome copy number. DsGFP injection was included as control (n = 15). (**B**) SBPV: DsVago (n = 16) was injected to silence *Bt*Vago, two days after injection, SBPV was injected to inoculate bees. Subsequently, RNA was collected post two days injection of SBPV for measuring viral genome copy number. DsGFP injection was included as control (n = 20). The means of each genome copy number were represented based on Log_10_ transformation and each bar errors were represented by standard error of mean. Different letters represent statistical significant difference of mean (*p* < *0.05*).

To further explore the possible role of *Bt*Vago during viral infections, we measured the amount of viral genome copy numbers after the silencing of *Bt*Vago. The amounts of IAPV showed no differences between treatments with dsGFP (n = 15) and dsVago (n = 9) (Fig. 4A). The amount of SBPV genome copy numbers also stayed constant between treatments dsGFP (n = 20) and dsVago (n = 16) (Fig. 4B).

### Silencing of *Bt*Hop temporarily increased the genome copy numbers of SBPV

**Figure 5:**
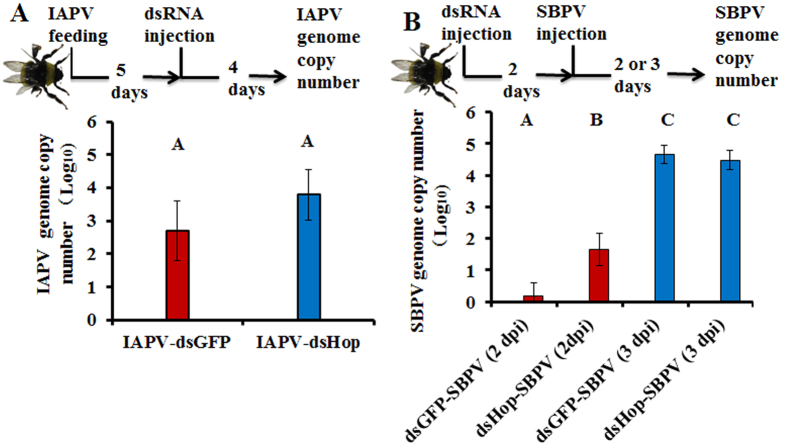
Genome copy numbers of viruses upon silencing of core JAK/STAT pathway gene: *Bt*Hop. (**A**) IAPV: The injection of IAPV causes extremely high and fast mortality, therefore, we used feeding as the inoculation method. IAPV was firstly ingested by fixed age adult bees, and then after five days later, dsRNA were injected to silence *Bt*Hop. DsGFP and ES (Elution buffer) were included as the controls. Subsequently, post four days injection of dsRNA, genome copy number of IAPV was measured. (**B**) SBPV: dsRNA were firstly injected to silence *Bt*Hop in age fixed adult bees. After two days later, we injected SBPV to infect bumblebees. Subsequently, post two and three days injection of dsRNA, genome copy number of SBPV was measured. At least 8 biological replicates were included in each treatment. The means of each genome copy number were represented based on Log_10_ transformation and each bar errors were represented by standard error of mean. Different letters represent statistical significant difference of mean (*p* < *0.05*).

To identify the involvement of the JAK/STAT pathway upon virus infections, we looked whether the silencing of *Bt*Hop could influence viral infections (Fig. 5). We detected the viral genome copy numbers upon the silencing of *Bt*Hop. The results revealed that the silencing of *Bt*Hop did not influence the amount of IAPV genome copy numbers compared with the dsGFP control (Fig. 5A). However, it significantly increased the amount of genome copy numbers of SBPV at two days post injection (dpi) but not at three dpi (t-test: t = −2.683, df = 19, *p* = *0.015*) (Fig. 5B). These results suggest that the JAK/STAT pathway plays a role against SBPV infection.

## Discussion

Dicer-2 belongs to the same DExD/H-box helicase family as the RIG-I-like receptors, which sense viral infection and mediate interferon induction in mammals^32^. The induction of core genes, especially *Dicer-2*, in the siRNA pathway by viral infection is detected in the honeybee^33^ and the bumblebee^24^. Studies from *Drosophila* and mosquito showed that Vago could be induced in a Dicer-2-dependent manner upon viral infection^7^,^15^,^32^. Conversely, in the current study, we observed that IAPV infection increased the expression of *Bt*Dicer-2, while the expression of *Bt*Vago was reduced. To the best of our knowledge, this is the first time to report the downregulation of *Vago/Vago-like* in insects upon viral infection. The downregulation of *Bt*Vago seems linked with IAPV rather than with *Dicer-2* since the infection of SBPV resulted in only the upregulation of *Bt*Dicer-2 but no change of *Bt*Vago. In *Drosophila*, Vago is induced after viral infections, such as DCV and Sindbis virus, but not from flock house virus^32^. All these evidences suggest that the expression of Vago (up, down, or constant) may be “case by case” dependent on the context of virus species and/or virus–host interactions.

Vago appears to be a cytokine that acts in a similar manner with mammalian interferon to induce the antiviral activity of the JAK/STAT pathway in neighboring cells^7^,^15^. The silencing of *Cq*Hop, but not *Cq*Dome, influenced the *Cq*Vago-induced suppression of WNV replication through the JAK/STAT pathway^7^. Thus, we tried to investigate whether *Bt*Vago played a role in the communication between the siRNA and JAK/STAT pathways upon infection with IAPV and SBPV. In our study, with the silencing of *Bt*Vago, it showed a reduced expression of *Bt*Dicer-2, but not *Bt*Hop, upon the infection of IAPV. Conversely, there was a reduced expression of *Bt*Hop, but not *Bt*Dicer-2, upon the infection of SBPV. These results indicate that the described association of Vago with JAK/STAT was noticed in bumblebees upon the infection of the avirulent virus SBPV. However, this association could not be proven when bees were infected with the virulent virus IAPV. To further verify the biological context of the downregulation of Vago to viral infections, we silenced *Bt*Vago to detect its impact on IAPV and SBPV infections. In contrast to our expectations it did not show any influence on virus genome copy numbers for both viruses. However, in other studies, the silencing of Vago or the mutation of Vago could lead to an increase of virus titers^7^,^32^. Nevertheless, in our experimental setup, it could indeed miss the correct time-points or sampling tissues (the lacking of information about viral titters in tissues rather than the whole body) to see the expected effect of the associations between RNAi (*Bt*Dicer-2) and JAK/STAT (*Bt*Hop) through *Bt*Vago upon infections of IAPV and SBPV in bumblebees. Thus, further studies are needed to elucidate more details on this aspect.

Considering that the JAK/STAT pathway in insects could be triggered by two ways, in a direct and or indirect (e.g. Vago) manner upon viral infection^7^,^8^,^9^, it is important to understand the role of this pathway upon different viral infections, especially, virulent and avirulent viruses. When we silenced *Bt*Hop we observed an increased genome copy number of SBPV at 2 dpi. The involvement of this pathway in IAPV infection is less clear since the same approach failed to detect any influence on IAPV genome copy numbers. IAPV exhibited an extremely virulent infection in the current setup; thus a temporally silencing of *Bt*Hop may not provide a significant effect on the influence of IAPV genome copy numbers if the JAK/STAT pathway is involved in combating against IAPV infection in bumblebee.

Taken all above evidences together, the role of *Bt*Vago may be different upon infection with the virulent virus IAPV and the avirulent virus SBPV. Indeed, we formulated speculation on the role of *Bt*Vago upon the infection with different types of viruses. One, in the case that the infection is under control by the host (i.e. the bees do not die from SBPV) *Bt*Vago may act in a similar manner as the described cross-talk bridge between the siRNA and the JAK/STAT pathways in mosquito^7^,^15^. Conversely, we also observed that the so-called cross-talk role of *Bt*Vago may be different upon the virulent virus infection (IAPV).

An important question to consider is whether this downregulation of *Bt*Vago upon IAPV infection is of interest to the host or/and the virus. On the one hand, it could be beneficiary to the virus via partially shutting off the *Bt*Vago communication between the siRNA and JAK/STAT pathways. On the other hand, it could also be beneficiary to the host because switching off the induction of the extra activities of JAK/STAT triggered by Vago may be able to open opportunities for other antiviral pathways. For instance, the activation of *Cq*Vago requires Relish2^15^ which is one of the components in the Imd antiviral pathway^34^. The downregulation of Vago could possibly be related with the increase of the activity of Imd in controlling viruses.

In conclusion, this project indicated that the possible role of Vago/Vago-like may vary between the infection with virulent (IAPV) and avirulent (SBPV) viruses in bumblebees. Upon SBPV infection, we see no effect on *Bt*Vago expression, but find evidence for the proposed interaction between Vago and the JAK/STAT pathway which could play a role in tempering SBPV infections. While upon IAPV infection, *Bt*Vago was down-regulated, potentially related with high virulence of this virus. Further studies are required to analyze this proposed role of Vago/Vago-like in viral virulence and in its association with antiviral immunities in bees.

## Methods

### Insects and viruses

Newly emerged workers were collected from the colonies of *B. terrestris* provided by Biobest NV (Belgium), and kept in micro-colonies fed with pollen and sugar water *ad libitum* in incubator under the condition of 29–31 °C, 60–65% relative humidity, and continuous darkness, for further experiments. Two bee viruses were chosen in this study based on their virulence in bumblebees: IAPV (virulent virus) presents an extremely fast replication and causes high mortalities of the bees within few days; SBPV (avirulent virus), its infection causing no mortality, replicates fast but slower compared to IAPV^24^. Colonies used in this study were screened to be free of IAPV and SBPV infections. In detail, ten bees from each colony were randomly collected and checked for the infection status of IAPV and SBPV by RT-PCR (Supplementary file: Table S2). The infection status of other common bee viruses (except IAPV and SBPV) in the bees used in this study was not clear, which needs to be pointed out since it could possibly influence the overall antiviral immunity of hosts.

IAPV and SBPV inocula were produced by propagating virus reference isolates in 50 white-eyed honeybee pupae and preparing a chloroform-clarified extract in PBS (10 mM phosphate buffer (pH 7.0)/0.02% diethyl dithiocarbamate) by following the protocol described in a previous study^35^. The contamination of other common bee viruses in these inocula, such as acute bee paralysis virus, Kashmir bee virus, SBPV or IAPV, Chronic bee paralysis virus, DWV, Varroa destructor virus-1, sacbrood virus and black queen cell virus, were determined by RT-qPCR and was less than 0.1%. The genome copy number of the IAPV and SBPV inocula used in this study were 2.34 × 10^10^ and 4.5 × 10^11^ per μl, respectively. To further analyze the number of viral particles, the inocula were measured through transmission electron microscopy with a standard protocol for negative staining by CODA-CERVA (Brussels, Belgium). The estimated number of particles were 1 × 10^6^ for both IAPV and SBPV inocula. The genome copy number of virus represent all the mRNA associated with virus, which could be virus expressed mRNAs or viral genome, while TEM is focused on viral particles (capsid). In addition to the difference in detection sensitivity of qPCR and TEM, the estimation of viral concentration from qPCR is normally higher than that from TEM.

### Viral inoculations

Bees were transferred into 50 ml tube and incubated on ice for ~20 min. Then, the unconscious bees were immediately injected with virus by the nano-injector. For IAPV injection, we used ~20 particles (in 5 μl solution) per bee and the amount of SBPV was ~200,000 particles (in 5 μl solution) per bee. PBS injected bees served as control. To maintain an accurate injection and avoid any leak of injection solutions, we chose the soft white-like cuticle between the 1^st^ and 2^nd^ segments as injection site and the injection process were strictly screened under microscope. The injected bees were immediately transferred back to micro-colonies with the same condition as described before. When IAPV feeding was required, bees with 5 hours starvation were transferred into a petri dish, and a liquid drop (in 20 μl solution) containing of an amount of 10^8^ particles (mixed with sugar water instantly before feeding) were ingested per bee. Only bees that directly and completely ingested the solution within one hour were put back to micro-colonies. The control treatment was followed with the same procedure but with PBS spiked sugar water.

### Gene silencing by dsRNA

A fragment of target gene was amplified by PCR with gene sequence specific primer plus T7 promoters (Supplementary file: Table S2). Then, these partial DNA templates of each gene were purified by E.Z.N.A. Cycle-Pure Kit (Omega, USA). The specificity of each template was checked by running the PCR products in an electrophoresis on 1.5% agarose gel and sequence confirmations for these templates (LGC genomics, Germany). Next, one microgram templates were used to synthesis dsRNA according to the guideline of MEGAscript RNAi Kit (Invitrogen, USA). The concentration and quality of each dsRNA were verified by Nanodrop and electrophoresis on 1.5% agarose gel. With the same procedure, a partial of GFP sequence (Supplementary file: S2) was used as template to synthesis dsGFP as negative control. For each gene silencing, a total of 20 μg (in a volume of 20 μl) of dsRNA were injected per bee, and same dose of dsGFP served as negative control for the effect of non-specific dsRNA. The injection of *Bt*Dicer-2, *Bt*Vago, and *Bt*Hop specific dsRNA showed a depletion of 59.6%, 67.6%, and 60.6% of its expression, respectively (Supplementary data, Figure S4).

### Sample preparation for exploring the possible role of *Bt*Vago

#### Samples with viral infections

Two days after injection of IAPV, the whole body of the bee was collected for RNA extraction (n = 10), the bees injected with PBS undergoing the same procedure (n = 10) was used as control. For IAPV infection through feeding, we collected bees after 9 days after IAPV feeding. The abdomen of each individual (n = 8) was used to extract RNA. PBS fed bees (n = 8) were used as controls. For SBPV, after 3 days injection, the abdomen of each bee was used for RNA isolation (n = 8), and the same treatment of PBS injection was used as control (n = 8). These time points used for viral inoculations or sample collections were based on the difference of viral virulence and viral titer dynamics^24^. The whole experiment was repeated twice.

#### Samples with silencing of *Bt*Vago or *Bt*Dicer-2 and viral infections

We first silenced the genes through injection of the sequence specific dsRNA, after two days, we inoculated bees with SBPV or IAPV by injection. Subsequently, post two days of SBPV injection and post 1.5 days IAPV infection, RNA for each group were collected. For group of SBPV, two treatments were applied, dsDicer-2-SBPV (n = 8) and dsVago-SBPV (n = 15). For groups of IAPV, two groups were involved: dsDicer-2-IAPV (n = 18) and dsVago-IAPV (n = 9). In each treatment, dsGFP controls were applied as the controls, respectively. These time points used for viral inoculations or sample collections were based on the difference of viral virulence and viral titer dynamics^24^. The whole experiment was repeated twice.

### Detection of viral genome copy number

To measure whether the silencing of *Bt*Hop and *Bt*Vago could influence the amount of IAPV and SBPV genome copy number, the relative viral genome copy numbers in each sample were evaluated based on the standard curves calculated by qPCR in previous study^24^: with cq values (x) and corresponding genome copy number (y: log_10_), for IAPV, the equation is y = −0.3017x + 8.8995 (R^2^ = 0.9997); for SBPV, the equation is y = −0.2926x + 9.4426 (R^2^ = 0.9996). The normalized genome copy number of each sample was represented by the ratio of the genome copy number calculated through the DNA standard curve and the normalization factor from the internal reference gene *PPIA*^35^.

### RNA isolation, cDNA, and qPCR

RNA isolations were applied by RNeasy Mini Kit (Qiagen, Germany) according to manufactures’ instruction with a small modification in sample collection. In detail, 1.5~2 ml RLT buffer were used to homogenize the lysed bumblebee tissues by mortar and the supernatants were centrifuged for three times to remove the tissue-debris pellet. Afterwards the steps were followed by the standard protocol of the kit. Genomic DNA was removed by treatment with TURBO DNA-free Kit (Ambion, USA). RNA quantity and quality were checked by Nanodrop and electrophoresis on 1.5% agarose gel. Two micrograms of RNA were used to synthesize the cDNA in a volume of 20 μl by SuperScript II Reverse Transcriptase (Invitrogen, USA) using oligo (dT) primers. To confirm that genomic DNA was completely removed we checked cDNA samples with exon spanning primers for RPL23 (Supplementary data Table S2). The cDNA should produce an amplicon of 143 bp while possible genomic DNA contamination would produce an extra amplicon of 452 bp. The qPCR was performed in a volume of 20 μl on a CFX96 Real-Time PCR Detection System (BioRad, USA) using GoTaq qPCR Master (Promega, USA). Each reaction was performed in duplicate and consisted of 10 μl of GoTaq qPCR Master mix, 1 μl of each primer (forward and reverse), and 8 μl of cDNA (from 100 times dilution of cDNA synthesized above). A delta Cq of less than 0.5 was applied for duplicates to pass the quality control. Few samples failed and were re-analyzed in duplicate following the same criteria. The amplification specificity of primers was checked by both electrophoresis of the RT-PCR products and analysis of the dissociation curve of qPCR. In addition, RT-PCR products amplified by these primers were sequenced in order to confirm their primers’ specificities.

### Data analysis

The data transformation and normalization of gene expression was followed as the framework of qBase^36^. First, relative quantities for each gene in each sample by comparing the *Cq* of a given sample with the average *Cq* across all samples for that gene was calculated, with taking into account of the amplification efficiency of that gene; next, the relative quantities of that gene was normalized by the relative quantities of the optimal reference gene (*PPIA*)^35^; finally, the relative expression of each gene was rescaled to the average expression level across all samples. The normality of all the data sets was tested by Shapiro-Wilk, then the mean comparisons of gene expressions and viral genome copy number (log_10_ transformed) were separated by independent samples t-test (for data sets that passed the normality test) or Mann-Whitney U test (for data sets that failed to pass the normality test) by SPSS statistics 22. Additionally, both t-test and Mann-Whitney U test were used for all the data sets to increase the power of the tests, while both tests showed consistent results of the significant level with each other. A significant level was indicated as *p* < *0.05*.

## Additional Information

**How to cite this article**: Niu, J. *et al*. Differential expression pattern of *Vago* in bumblebee (*Bombus terrestris*), induced by virulent and avirulent virus infections. *Sci. Rep.* **6**, 34200; doi: 10.1038/srep34200 (2016).

## Supplementary Material

Supplementary Information
