# Supplementary material for: Differential expression pattern of Vago in bumblebee (Bombus terrestris), induced by virulent and avirulent virus infections
Source: Sci Rep. 2016 Sep 29;6:34200. doi: 10.1038/srep34200 (PMC5040954; doi:10.1038/srep34200)
Supplement: Supplementary Information [file srep34200-s1.pdf]

Title:

Differential expression pattern of *Vago* in bumblebee (*Bombus terrestris*), induced by virulent and avirulent virus infections

Jinzhi Niu[1,2], Ivan Meeus [2], Guy Smagghe[1,2,*]

[1]Key Laboratory of Entomology and Pest Control Engineering, College of Plant Protection, Southwest University, Chongqing 400716, China

[2] Department of Crop Protection, Faculty of Bioscience Engineering, Ghent University, Coupure Links 653, B-9000 Ghent, Belgium

*Corresponding author: guy.smagghe@ugent.be

Supplementary data

Figure S1: The eight conserved cysteine residues in VWC domain of *Bt*Vago compared with *Cq*Vago

```
Culex           MKTFGVIILLVCCAIGGS--------LAAEAVLQNAEHPDYPGKCYDEGTQTVVAPLESA
Bombus          MKLVPVLLLVAAIVFAAEEKKEEERPKTFRRLIPADVLRDFPGMCFASTRCATIEPTKSW
                ** . *::*:.. .:...         : . ::      *:** *: .   :.: * :*

Culex           KLPKSCTKVFCSTN------LSLTYTTCGSVLVNDPHCEKIE-QDLTKDFPECCHKYKCE
Bombus          DLTPFCGRSTCVPADDNSGRLFELVEDCGPLPKANPKCKLSDKTNKTATFPDCCPIFECE
                .*   * :  *         *      ** :   :*:*:  :  : *  **:**  ::**

Culex           LEGVVTYH-------------------------
Bombus          EGAKLEYPEIPTLPPPTEIVETEKTPEATPAKA
                  . : *
```

Figure S2: Predicated signal peptide cleavage site of *Bt*Vago

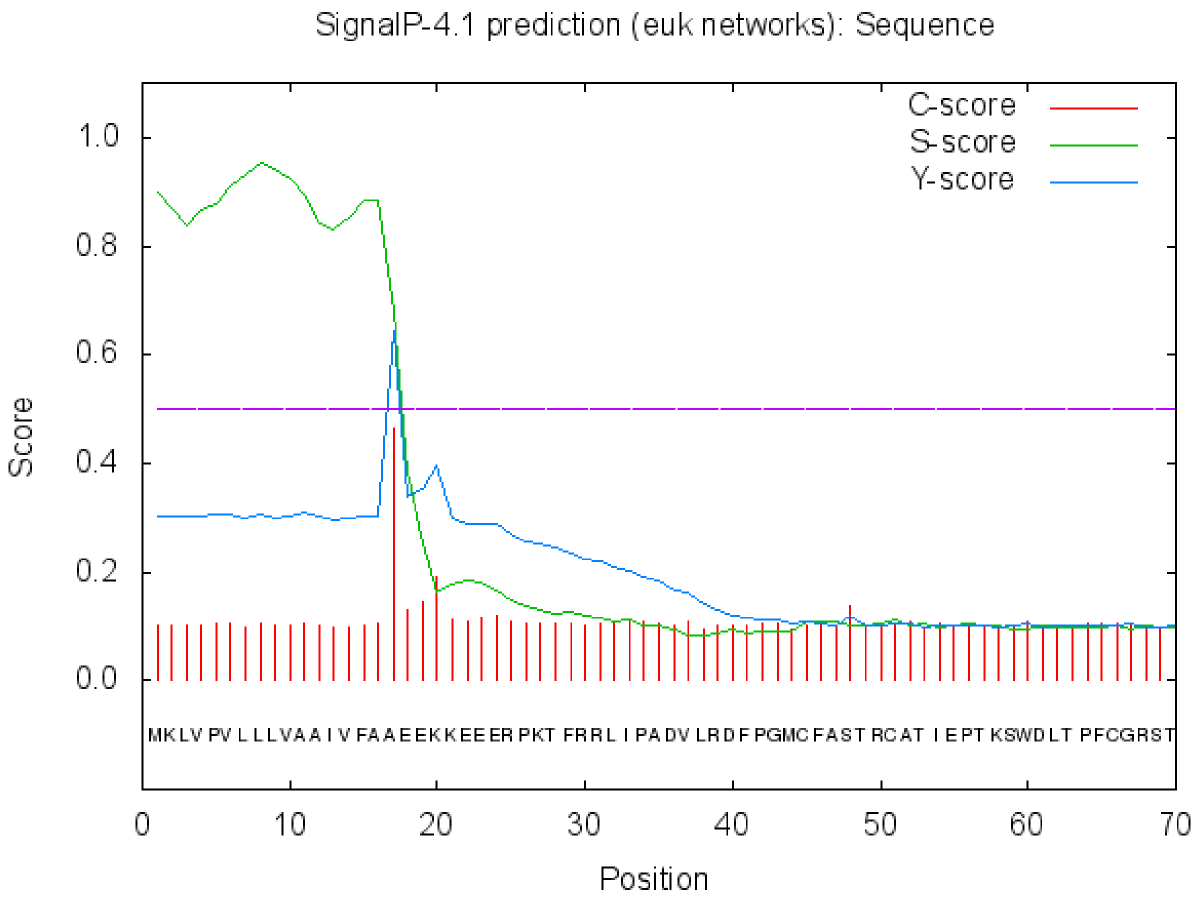

Figure S3: The expression of *Bt*Dicer-2, *Bt*Vago and *Bt*Hop upon the silencing of *Bt*Dicer-2 (A) or *Bt*Vago (B)

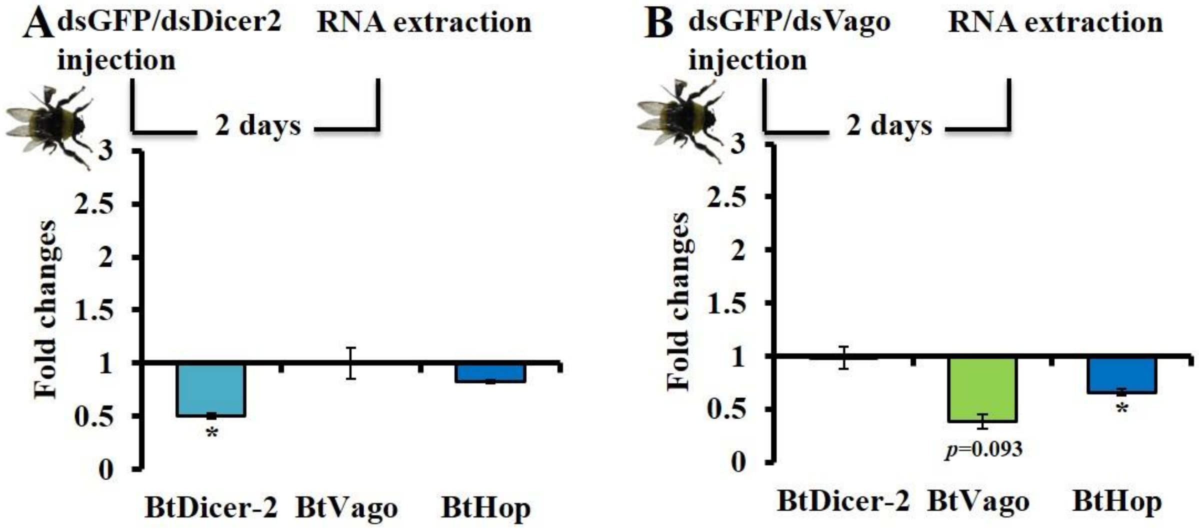

Figure S4: Silencing of *Bt*Dicer-2 (A), *Bt*Vago (B), and *Bt*Hop (C) through injections of dsRNA

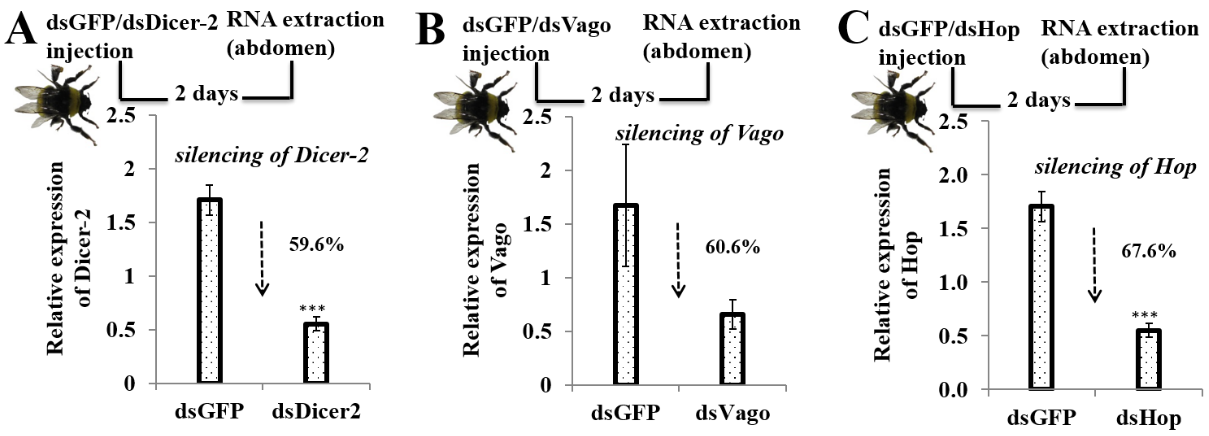

Table S1: Predicted NF-κB biding site in *Bt*Vago promoter region

Factor name	Start position	End position	Dissimilarity	String	RE equally	RE query
RelA [T00595]	438	448	12.115729	TCTAACGAAGC	0.02146	0.01273
GATA-1 [T00305]	735	742	4.138263	CACGATAA	0.54932	0.69044
GATA-1 [T00305]	925	932	1.520654	TTATCATT	0.30518	0.65047
GATA-1 [T00305]	1609	1616	4.052395	TCGGATAA	0.54932	0.69044
GATA-1 [T00305]	1896	1903	1.682543	TTATCACA	0.30518	0.65047
GATA-1 [T00267]	735	743	8.557309	CACGATAAT	0.06866	0.14082
GATA-1 [T00267]	924	932	4.979757	TTTATCATT	0.18311	0.36798
GATA-1 [T00267]	1609	1617	3.166737	TCGGATAAG	0.11444	0.16757
GATA-1 [T00267]	1895	1903	3.40581	CTTATCACA	0.10681	0.17821

Table S2: Primers used in this study

Name	Primer sequence	Amplicon length	Purpose
qPCRVagoF2	TGTTACCCTTCAACGCAATTC	194	QPCR for Vago
qPCRVagoR2	ACAGATTCCGAAACGCTGAT		
Vago-T7dsF2	TAATACGACTCACTATAGGGAGACCTAGTCCCGGAAGTCGAGA	418	dsRNA synthesis for Vago
Vago-T7dsR2	TAATACGACTCACTATAGGGAGAGTACGTACGAATTACAAGATCAACT		
qPCRHopF3	TGGCACAATGTGTCTCATCTT	186	QPCR for Hop
qPCRHopR3	GAGGTACACAACGAGGTCCAG		
Ds2ndHOP-F4	TAATACGACTCACTATAGGGAGATGTCCTTTGTTTCTGCTCTGGA	374	dsRNA synthesis for Hop
Ds2ndHOP-R4	TAATACGACTCACTATAGGGAGATGACTGTCCTTCAGAATCTTGGA		
q-Ri-dicer2-F2	TGGTCAAAACATCAAGAACAACCA	166	QPCR for Dicer-2
q-Ri-dicer2-R2	GATCGGGGCCATACGAACAT		
C-dsDicer2-2nd-F4	TAATACGACTCACTATAGGGAGAGCGAAGGTGTCACCAAATGT	437	dsRNA synthesis for Dicer-2
C-dsDicer2-2nd-R4	TAATACGACTCACTATAGGGAGAGGGTGTGTAAAGGCCTGCAA		

S1: Conserved proteins sequences used to construct phylogenetic tree of Vago

>Bombus_terrestris

MKLVPVLLLVAAIVFAAEEKKEEERPKTFRRLIPADVLRDFPGMCFASTRCATIEPTKSWDLTPFCGRSTCVPADDNSGR

LFELVEDCGPLPKANPKCKLSDKTNKTATFPDCCPIFECEEGAKLEYPEIPTLPPPTEIVETEKTPEATPA

>PREDICTED:_uncharacterized_protein_LOC411622_Apis_mellifera.

MKFAPILLFVIAIVFAAEE-KEEERPKTFRRLIPADVLRDFPGMCFASTRCATIEPTKSWELTPFCGRSTCVPADDNSGR

LFELVEDCGPLPKANPKCKLSDKTNKTAAFPNCCPIFECEEGAKLEYPEIPTLPPPTEIIETEKTSEEVPT

>PREDICTED:_uncharacterized_protein_LOC105185794_Harpegnathos_saltator.

MEHAFVLLFTTIVFIAIADEKEEERPKTFRRLIPADVLRDFPGMCFASTKCATIEPTKTWELSPFCGRSTCVPADDNSGR

LFELVEDCGPLPKANPKCKLSDKTNKTASFPDCCPIFECEDGAKLEYPEIPTLPPPTEIEASEITAAPKV-

>PREDICTED:_uncharacterized_protein_LOC100878806_Megachile_rotundata.

MKLATILLLVVAVVFAAEE-KEEERPKTFRRLIPADVLRDFPGRCFASTKCATIEPTKSWDLTPFCGRSTCVPADDNSGR

LFELVEDCGPLPKANPKCKLSEKTNKSAPFPDCCPIFECEDGAKLEYPEIPTLPPPTEIVETEKTPEAAPA

>PREDICTED:_uncharacterized_protein_LOC105623255_Atta_cephalotes.

MKLVFALLCVAVAFVAAEDAQVQERPKTFRRLIPADVLRDFPGMCFASTKCATIEPTKSWELSPFCGRSTCVPADDNSGR

LFELVEDCGPLPKANPKCKLSDKTNKTASFPECCPIFECEDGAKLEYPEIPTLPPPSDEDAAKAQPETPKP

>PREDICTED:_uncharacterized_protein_LOC105282831_isoform_X2_Cerapachys_biroi.

MAFLLPHPCASSSV---------KIERNFR--------ENFPGMCFASTKCATIEPTKSWQLAPFCGRSTCVPADDNSGR

LFELVEDCGPLPKANPKCKLSDKTNKTASFPDCCPIFECEDGAKLEYPEIPTLPTPTED-AAKGEPAAAAP

>PREDICTED:_uncharacterized_protein_LOC105148313_Acromyrmex_echinatior.

MKLVFALLCVAAVFVATQDAQVQERPKTFRRLIPADVLRDFPGMCFASTKCATIEPTKSWELAPFCGRSTCVPADDNSGR

LFELVEDCGPLPKANPKCKLSDKTNKTASFPECCPIFECENGAKLEYPEIPTLPPPSED-AAKAQPEAPKP

>PREDICTED:_uncharacterized_protein_LOC105455873_Wasmannia_auropunctata.

MKTVFALLCVAVAFVATQGAQEQERPKTFRRLIPADVLRDFPGMCFASTKCATIEPTKSWELAPFCGRSTCVPADDNSGR

LFELVEDCGPLPKANPKCKLSDKTNKTSSFPDCCPIFECEDGAKLEYPEIPTLPPPSGEDAAAAQPQAPKP

>PREDICTED:_uncharacterized_protein_LOC105258902_Camponotus_floridanus.

MKLIFILLCAAVAFVAAEGTTEQERPKTFRRLIPADVLRDFPGMCFASTKCATIEPTKTWELSPFCGRSTCVPADDNSGR

LFELVEDCGPLPKANPKCKLSEKTNKTASFPDCCPIFECEEGVKLEYPEIPTLPPPTEAEAANVPPEATPK

>PREDICTED:_uncharacterized_protein_LOC105561162_Vollenhovia_emeryi.

MKTIFVLLCVAVAFVAAQDVQEQERPKTFRRLIPADVLRDFPGMCFASTKCATIEPTKSWELAPFCGRSTCVPADDNSGR

LFELVEDCGPLPKANSKCKLSDKTNKTASFPDCCPIFECEDGAKLEYPDIPTLPPPSDAI-AKAQPETPKP

>PREDICTED:_uncharacterized_protein_LOC103577816_Microplitis_demolitor.

NKYFAIVLLVAAVVVAEEE----ERPKTFRRLIPADVLRDFPGMCFASTKCATVEPTKSWELSPFCGRSTCVPSDDDSGR

LFELVEDCGPLPKANPKCKLSEKTNKTASFPDCCPIFECEEGAKLEYPEIPTVPPPPS--EAEAKPEVAKV

>PREDICTED:_uncharacterized_protein_LOC105197444_Solenopsis_invicta.

MKI-VFALCVAVAFVAANA-EEQERPKTFRRLIPADVLRDFPGMCFASTKCATIEPLKSWELSPFCGRSTCVLADDNSGR

LFELVEDCGPLPKANPKCKLSDKTNKTASFPDCCPLFECEEGAKLEYPEIPTLPPPSGVDTAKAQPEAPQS

>PREDICTED:_uncharacterized_protein_LOC100113619_Nasonia_vitripennis.

FSLALVAIVAVVAVHAAEEKKDEDRPKTYRRLIPADVLRDFPGMCFASTKCATIEPKQSWDLAPFCGRSTCVPADDNSGR

LFELVEDCGPLPKANPKCKLSEKTNKTAPFPDCCPVFECEDGAKLEYPEIPTLPPPTAEEIAKAAAAGKPA

>PREDICTED:_LOW_QUALITY_PROTEIN:_uncharacterized_protein_LOC105428984_partial_Pogonomyrmex_barbatus.

WKLTYLFTISEVLFFDSFLSGFYPNKRKCHLFVLFHYIVDFPGTCFASTKCATIEPGKSWELSPFCGRSTCVSADDNSGR

LFELVEDCGPLPKANPKCKLSDKTNKTASFPECCPIFECEDGATLEYPDIPTLPPPTED-AAKAQPEAPKP

>PREDICTED:_uncharacterized_protein_LOC105268204_Fopius_arisanus.

MKYVVAILFIAALVVAED--KDEERPKTYRRLIPADILRDFPGMCFASTKCATVEPGKTWDLSPFCGRSTCVPAEDNSGR

LFELVEDCGPLPKENPKCKLSEKTKKTSPFPDCCPIFECEEGAKLEYPEIPTIPPPELVEPTETTTAKA--

>conserved_hypothetical_protein_Pediculus_humanus_corporis.

MKFLIVFLIGFGAFCFGQETETTERPKVFRRLIPADVLRDFPGMCFASTKCTTVEPGKSWDLTPFCGRSTCVVAEEQAGR

LLELVEDCGPLPKANPKCKLSEKTNKTAPFPDCCPIFECEKGVKLEYPELPTVPPPTDKKKA---------

>CG31997_isoform_A_Drosophila_melanogaster.

MSFHTLILTAFTVSLCAEQKKSDAGERIFKRLIPADVLRDFPGMCFASTRCATVEPGKSWDLTPFCGRSTCVQNEENDAK

LFELVEDCGPLPLANDKCKLTEKTNKTASFPYCCPIFTCDPGVKLEYPEIGKDNDKKNSE-----------

>PREDICTED:_uncharacterized_protein_LOC105220319_Bactrocera_cucurbitae.

MKAFLVVACALFVCVAAQDDTSQTNSKIYKRLIPADVLRDFPGMCFASTRCATVEPTKTWELTPFCGRSTCVQNDENPAK

LLELVEDCGPLPLANEKCKLTEKTNKTAPFPYCCPIFTCEPGVKLEYPEIPKEEAKKE-------------

>uncharacterized_protein_LOC100167365_precursor_Acyrthosiphon_pisum.

MDYRAVVLGVAAIVLSADEKTPAPEARIYRRLIPADVLRDFPGLCFASTKCATVEPGHTWELSPFCGRSTCVQGE-GTDR

LLELVEDCGPYPKSNPKCKLSEKTNKTASFPECCPVFDCEPGVKLEYPEVTVVENPESSDDSTTTTSTEKP

>PREDICTED:_uncharacterized_protein_LOC655864_Tribolium_castaneum.

MKV-FLVLALCLAVAAAEDKKEEERPKTFKRLIPADVLRDFPGMCFASTKCATIEPGKTWELHPFCGRSTCVVSEDKPPR

LLELVEDCGPLPLANPKCKLEEKTNKTAAFPDCCPVFKCEEGAKLEYPEIPTVAPVPE--ASSTTPKA---

>hypothetical_protein_KGM_07285_Danaus_plexippus.

MKY-LIVLAIIAFSYAADD-KDDERPKTFRRLIPADVLRDFPGLCFASTRCATVEPGNTWDLAPFCGKSTCVVSEDTPPR

LLELVEDCGPLPIANPKCKLTDKTNKTAPFPDCCPIFTCEDGVKLEYPELPTPPEEEKKEKS---------

>PREDICTED:_uncharacterized_protein_LOC105665112_Ceratitis_capitata.

MKASLVIFVALFLYGAAQDAKNSSELKIYKRLIPADVLRDFPGMCFASTRCATVEPTKTWELTPFCGRSTCVQNEDNPSK

LLELVEDCGPLPLANEKCKLTGKTNKTAPFPYCCPIFICEPGIKLEYPEVPREELNKE-------------

>unknown_Manduca_sexta.

MKYLVVVAVFALAFAAEEKE---DIPKTYKRLIPADVLRDFPGTCFASTRCATVEPGNTWELSPFCGRSTCVLSEDHPPR

LLELVEDCGPLPLANPKCKLTDKTNKTAPFPGCCPIFTCEEGAKLEYPELPTPPPEGE--KAEEKPKKA--

>similar_to_CG31997_Papilio_xuthus.

MKYLFVLAVFALAFAAEEE-KDGERPKTFRRLIPADVLRDFPGLCFASTRCATVEPGNSWDLAPFCGRSMCVVSEDTPPR

LLELVEDCGPLPLANPKCKLTDKTNKTAPFPGCCPIFTCEDGVKLEYPELPTAAPEEEK-KAEEKPKA---

>conserved_hypothetical_protein_Culex_quinquefasciatus.

GVFVVAAALCVANVFAADEPTKDEGVKIYKRLIPADVLRDFPGMCFASTRCATIEPGKSWDLAPFCGRSTCVVSESNPAQ

LLELVEDCGPLPLANDKCKLTDKTNKTAPFPYCCPKFTCEPGVKLEYPEIKPSDASEEKK-----------

>AAEL000445-PA_Aedes_aegypti.

GVLIAATLCVANVFAADEAPTDEDTVKIYKRLIPADVLRDFPGMCFASTRCATIEPGKSWDLAPFCGRSTCVVSETNPSQ

LLELVEDCGPLPLANDKCKLTDKTNKTAPFPYCCPKFTCEPGVKLEYPDVKGPEAAPEDD--KKN------

>PREDICTED:_uncharacterized_protein_LOC105381502_Plutella_xylostella.

MKY-LVAFAVFALAFAAEEKAEEERPKTFRRLIPADVLRDFPGMCFASTRCATVEPGNAWDLSPFCGRSTCVVSEDEPPR

LLELVEDCGPLPLANDKCKLTDKTNKTAPFPGCCPVFTCTDGAKLEYPELPTPPPEGE----EKKPEEEKP

>PREDICTED:_uncharacterized_protein_LOC101888253_Musca_domestica.

MKFFAVFLLVACVYGAATDKTKEEAPKIYKRLIPADVLRDFPGMCFASTRCATVEVGKSWELTPFCGRSTCVQNEEDPSK

LLELVEDCGPLPLANDKCKLTEKTNKTAAFPYCCPVFTCEPGVKLEYPEAVKETKKE--------------

>AGAP002085-PA_Anopheles_gambiae_str._PEST.

MRSFTVVAVLALYVQAADEATKDAAPKTYKRLIPADVLRDFPGMCFASTKCATFEPGQYWDLTPFCGRSTCVLSDDAQPR

LLELVEDCGPLPLANDKCKLTEKTNKTAPFPACCPTFTCEPGAKLEYPEIKTAPESTSEQN----------

>hypothetical_protein_YQE_10125_partial_Dendroctonus_ponderosae.

--MCFASTKCATVEPGKTWELYPFCGRSTCVVSEDQPPR

LLELVEDCGPLPLANEKCKLEEKTNKTAPFPACCPEFKCEAGAKLEYPEIPTVAPVPEDASTTTAKSA---

>PREDICTED:_uncharacterized_protein_LOC101741978_Bombyx_mori.

MKYLIVLAVFALAFAAEEKE---ERPKTFRRLIPADVLRDFPGMCFASTRCITVEPGNTWELSPFCGRSTCVVSEDQPPR

LLELVEDCGPLPLTNPKCQLTEKTNKTAPFPGCCPIFTCEEGAKLEYPELPTPPPEDK--KAEEKPKA---

>unknown_Culicoides_sp._LJH-2002.

MRFPFVFFVIVSLSFASET-KNDDGIKVYKRLIPADVLRDFPGVCFASTKCAMFEPGKQWDLKPFCGRSTCVTPEDGSSR

LLELVEDCGPLPIANDKCKLTEKTNKTADFPYCCPQFACQDGAKLEYPEVKTSSA----------------

>hypothetical_protein_DAPPUDRAFT_214640_Daphnia_pulex.

MKFILCFSLLFVLVASVE-----ITPKTLTREIKADVLRDFPGVCYASTQCRTFKENEEWDLKPFCGKSICIKGA--DGI

LKERVSDCGPPAKANPECKV--NANATLPYPNCCPVYDCAPGVQLEFPDIPVA------------------

>single_VWC_domain_protein_1_Litopenaeus_vannamei.

MKF-LLIACLGSLVFAQQGPADLQGPGPFVRDLKADVLRDFPELCFSSTNFRLFLENQSWSLFPFCGKAECVKSG---AD

YIERVHDCGPQPKNAEACTILQRNDTILEYPSCCPKYVCPDGVTLEYPEIQKQNQAALQAAAAAREAAGPQ

>Chain_A_Structure_Of_A_Chordin-Like_Cysteine-Rich_Repeat_(Vwc_Module)_From_Collagen_Iia.

-------------YVEFQEA----------------------GSCVQDGQ--RYNDKDVWKPEP-CRICVCDTGTLCDDI

ICEDVKDC---------------LSPEIPFGECCPI--CPA-------DLAAAA-----------------

S2: Sequence of dsGFP used as non-specific dsRNA

>dsGFP fragment 455 bp

TGATCGCGCTTCTCGTTGGGGTCTTTGCTCAGGGCGGACTGGGTGCTCAGGTAGTGGTTGTCGGGCAGCAGCACG
GGGCCGTCGCCGATGGGGGTGTTCTGCTGGTAGTGGTCGGCGAGCTGCACGCTGCCGTCCTCGATGTTGTGGCG
GATCTTGAAGTTCACCTTGATGCCGTTCTTCTGCTTGTCGGCCATGATATAGACGTTGTGGCTGTTGTAGTTGTACT
CCAGCTTGTGCCCCAGGATGTTGCCGTCCTCCTTGAAGTCGATGCCCTTCAGCTCGATGCGGTTCACCAGGGTGTC
GCCCTCGAACTTCACCTCGGCGCGGGTCTTGTAGTTGCCGTCGTCCTTGAAGAAGATGGTGCGCTCCTGGACGTAG
CCTTCGGGCATGGCGGACTTGAAGAAGTCGTGCTGCTTCATGTGGTCGGGGTAGCGGCTGAAGCACTGCACGCCG
TA
